# Lycopene from two food sources does not affect antioxidant or cholesterol status of middle-aged adults

**DOI:** 10.1186/1475-2891-3-15

**Published:** 2004-09-15

**Authors:** JK Collins, BH Arjmandi, PL Claypool, P Perkins-Veazie, RA Baker, BA Clevidence

**Affiliations:** 1 USDA, ARS, South Central Agricultural Research Laboratory, Lane, OK, USA; 2 Dept. Nutritional Sciences, Oklahoma State University, Stillwater, OK, USA; 3 Dept. Statistics, Oklahoma State University, Stillwater, OK, USA; 4 Retired, USDA, ARS, Citrus and Subtropical Products Laboratory, Winter Haven, FL, USA; 5 USDA, ARS, Diet and Human Performance Laboratory, Beltsville, MD, USA

## Abstract

**Background:**

Epidemiological studies have reported associations between reduced cardiovascular disease and diets rich in tomato and/or lycopene. Intervention studies have shown that lycopene-containing foods may reduce cholesterol levels and lipid peroxidation, factors implicated in the initiation of cardiovascular disease. The objective of this study was to determine whether consumption of lycopene rich foods conferred cardiovascular protection to middle-aged adults as indicated by plasma lipid concentrations and measures of *ex vivo *antioxidants.

**Methods:**

Ten healthy men and women consumed a low lycopene diet with no added lycopene (control treatment) or supplemented with watermelon or tomato juice each containing 20 mg lycopene. Subjects consumed each treatment for three weeks in a crossover design. Plasma, collected weekly was analyzed for total cholesterol, high density lipoprotein cholesterol (HDL-C) and triglyceride concentrations and for the antioxidant biomarkers of malondialdehyde formation products (MDA), plasma glutathione peroxidase (GPX) and ferric reducing ability of plasma (FRAP). Data were analyzed using Proc Mixed Procedure and associations between antioxidant and lipid measures were identified by Pearson's product moment correlation analysis.

**Results:**

Compared to the control diet, the lycopene-containing foods did not affect plasma lipid concentrations or antioxidant biomarkers. Women had higher total cholesterol, HDL-C and triglyceride concentrations than did the men. Total cholesterol was positively correlated to MDA and FRAP while HDL-C was positively correlated to MDA and GPX. GPX was negatively correlated to triglyceride concentration.

**Conclusions:**

The inclusion of watermelon or tomato juice containing 20 mg lycopene did not affect plasma lipid concentrations or antioxidant status of healthy subjects. However, plasma cholesterol levels impacted the results of MDA and FRAP antioxidant tests.

## Background

Watermelons and tomatoes are good sources of the carotenoid lycopene [1,2]. However, bioavailability of lycopene is not directly related to plant content, and depends in a large part upon plant matrix effects. In tomatoes, heat processing and homogenization breaks protein-carotenoid complexes, releases lycopene from cell wall linkages and improves human uptake of this compound [3-6], while heat processing is not necessary for adequate uptake of lycopene from watermelon juice [7]. Extracts of both foods exhibit antioxidant activity *in vitro *and function is attributed to lycopene since isolated lycopene demonstrates strong oxygen and peroxy radical scavenging properties [8-10].

Recent epidemiological studies have linked reductions in risks of cardiovascular disease with diets rich in lycopene containing foods. These reductions in risk have been primarily attributed to the antioxidant properties of lycopene [11,12]. Improved antioxidant parameters of lymphocytes have been reported in clinical trials that supplemented diets with 16.5 mg and 40 mg /day of lycopene from tomato puree and tomato juice, respectively [13,14]. Other clinical trials have shown reductions in low-density lipoprotein (LDL) oxidation resulting from lycopene supplementation [15-17]. LDL contains unsaturated fatty acids and can be oxidized by free radicals and peroxidizing agents. Since lycopene is primarily attached to LDL in plasma, it may protect against atherosclerosis through inhibition of lipid peroxidation and foam cell production [12,18].

Other studies have assessed response of plasma lipids to lycopene-rich diets. In one study, six healthy men were supplemented with 60 mg/day for three months with tomato lycopene (LycoRed) with a 14% reduction in LDL-C and no change in HDL-C [19]. Researchers concluded that lycopene was involved in controlling cholesterol synthesis and found the same results in a macrophage cell study [19]. It is not known if other lycopene containing foods can act ex *vivo *as antioxidants or alter cholesterol levels.

The objectives of this study were to compare the ability of two lycopene containing foods, tomato and watermelon to provide cardiovascular protection to middle-aged adults by measuring changes in cholesterol levels and antioxidant ex *vivo *biomarkers.

## Methods

### Experimental Design

Samples for this study came from a larger study, which has been reported in detail [7]. This study was a diet-controlled, repeated measures crossover design with ten healthy non-smoking subjects, five men (average age 49 years) and five women (average age 51 years) recruited from the Beltsville, MD area (Table 1). In addition to a base diet, which provided 34% of energy from fat and minimal amounts of lycopene, subjects were randomly assigned to receive three dietary treatments for 3 weeks each: 1) control (no added lycopene); 2) 20.1 mg lycopene per day from watermelon juice; and 3) 18.4 mg lycopene per day from tomato juice. All subjects followed a low-lycopene diet for two weeks before the first treatment and during the four-week washout periods between treatments. Total study duration was 19 weeks. During treatment periods, all meals were prepared and consumed Monday through Friday at the Beltsville Human Nutrition Research Center's Human Studies Facility, and weekend meals were packed for off-site consumption. Blood was drawn from fasted subjects before treatment (the day before the start of study and on the first day of the study), prior to treatment and weekly during treatment. Plasma was separated from whole blood by centrifugation and stored at -80°C until analyzed for cholesterol and antioxidant activity.

**Table 1:** Description of human clinical study participants.

Gender	N	Age (Range) yr	BMI (Range) Kg/m^2^
Men	5	49 (43–68)	26.3 (23.0–29.5)
Women	5	51 (35–63)	29.1 (23.5–34.5)

### Juice Treatments

Watermelon juice for the study was prepared at a pilot plant at the USDA Citrus and Subtropical Products Laboratory, Winter Haven, FL without heat treatment as previously described [7]. Canned commercial tomato juice (Campbell's, Camden, NJ) was used for the tomato intervention. Juices were analyzed for carotenoid content using established extraction procedures with reversed phase HPLC with photo diode array detector (Waters Corp, Franklin, MA) [7]. For watermelon treatment, subjects were given one bottle of juice (260 g each) at breakfast, lunch and dinner, which provided daily totals of 20.1 mg lycopene, 0.90 mg phytoene, 0.45 mg phytofluene and 2.5 mg beta carotene. The juice contained 94% *trans *lycopene and 6% *cis *isomers, primarily 5-*cis *and 13-*cis *with minimal amounts of other *cis *isomers [7]. For tomato juice treatment, subjects were given one serving (122 g each) at breakfast and dinner, which provided daily totals of 18.4 mg lycopene, 2.1 mg phytoene, 1.1 mg phytofluene and 0.6 mg beta carotene with 89% of the lycopene as *trans *lycopene and 10.8% *cis *isomers, primarily identified as 5-*cis*, 9-*cis*, 13-*cis*, and 15-*cis*, and minimal amounts of other *cis *isomers [7].

### Cholesterol Analysis

Plasma samples were thawed on ice for four hours then mixed by vortexing, prior to preparing for assays. Serum total cholesterol and triglyceride concentrations were determined enzymatically using kits from Roche Diagnostics (Sommerville, NJ). Serum HDL-cholesterol was determined by a direct method (Unimate HDL Direct ; Roche Diagnostics, Indianapolis, IN) that utilizes the combined action of polymers, polyanions, and detergent to solubilize cholesterol from HDL but not from VLDL, LDL, and chylomicrons as previously described [20]. Analysis was performed on a Cobas-Fara II Clinical Analyzer (Montclair, NJ) using commercially available calibrators and quality control standards (Roche Diagnostics, Indianapolis, IN).

### Plasma Glutathione Peroxidase Assay

Plasma from subjects was analyzed for plasma glutathione peroxidase using an ELISA kit (OXIS Internatl., Portland, OR). Two replicates per sample of 20 μl of plasma were diluted 1:25 with TRIS-HCl buffer then pippetted into pre-coated polyclonal antibodies microplate wells specific for human plasma glutathione peroxidase (GPX). The amount of enzyme present was determined by reaction with para-nitrophenyl-phosphate and was read using a microplate reader at 405 nm (Elx 808 Ultra Microplate Reader, Bio-Tek Instruments Inc., Winooski, VT). The concentration of plasma GPX was determined from a standard curve for each plate using five dilutions of GPX standard.

### Plasma lipid peroxidation

Malondialdehyde compounds were determined colorimetrically using a commercial kit specific for measuring free and total malondialdehyde compounds (OXIS Internatl., Portland, OR). Two replicates per sample of 210 μl of plasma were added to each test tube with 11 μl of 500 mM butylated hydroxytoluene and 5.3 μl of concentrated hydrochloric acid. Tubes were capped, mixed then incubated at 60°C for 80 minutes, cooled to room temperature and 680 μl of N-methyl-2-phenylindole in acetonitrile was added. Then tubes were mixed, and centrifuged at 13,000 g for 5 minutes. New tubes were prepared and 660 μl of clear supernatant was added with 115 μl of concentrated HCl. Tubes were capped, mixed and incubated at 45°C for 60 minutes. Samples were centrifuged at 13,000 g for 5 min and the supernatant was read on a spectrophometer at 575 nm. Concentration of samples was determined using a five point standard curve.

### Ferric reducing ability of plasma assay

This assay was conducted according to previously published methods [1]. In brief, three reagents were used: 1) sodium acetate, acetic acid buffer (pH 3.6); 2)10 mmol/L solution of 2, 4,6-tripyridyl-s-triazine in a 40 mmol/L solution of hydrochloric acid (Sigma, St. Louis, MO); and 3) 20 mmol/L solution of ferric chloride hexahydrate prepared in double deionized water. The FRAP reagent was prepared daily with 25 ml of reagent one, 2.5 ml reagent two and three that were heated to 37°C before using [21]. The assay was conducted with 10 uL of plasma that was diluted with 30 μl of ddi water. Sample was added to reagent in cuvettes with an autosampler and then read on a COBAS FARA II spectrofluorometric centrifugal analyzer (Roche, Montclair, NJ) at 593 nm at four minutes. FRAP values were determined from a five point curve using a trolox (vitamin E analog) standard. Standard curves were run after every 90 samples.

Experimental procedures for the clinical trial were approved by the Institutional Review Board at the Johns Hopkins University Bloomberg School of Hygiene and Public Health; subjects gave their written informed consent to participate. The plasma cholesterol and antioxidant studies were approved by the Institutional Review Board at Oklahoma State University, Stillwater, OK. Data were analyzed using Proc Mixed Procedure and mean separation was performed using LSMEANS, correlation analysis was performed using Spearman's Correlation Coefficient Analysis (SAS Statistical Analysis Software, version 8.2, SAS Institute, Cary, NC).

## Results

Because there were significant four way interactions with gender × intervention period × treatment × weeks with MDA, FRAP, GPX and cholesterol analysis, trends by treatment, intervention period or week of treatment were not seen. Supplementing the diet with 20 mg/day of lycopene of either food did not change the plasma antioxidant status of the subjects and values ranged from 0.66–2.20, 540–1094, and 1296–2596 :mol/L for MDA, FRAP and GPX respectively. These levels are similar to levels reported for healthy subjects in other studie [22,23].

**Figure 1:**
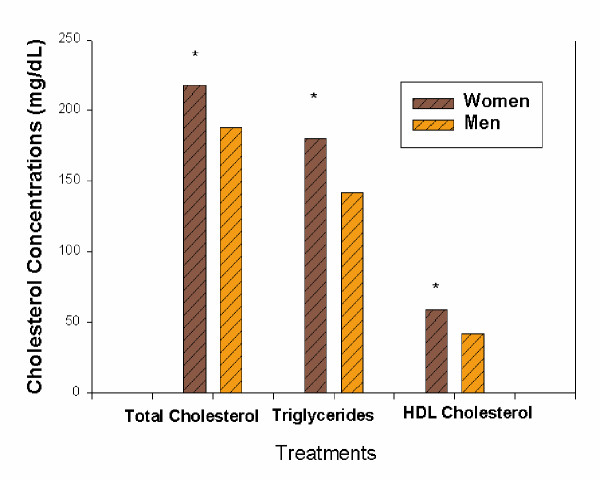
Mean plasma total cholesterol, triglycerides, and high density lipoprotein (HDL) cholesterol (mg/dl) of 5 men and 5 women supplemented for 3 week with no lycopene (control), 20 mg lycopene from watermelon juice and 20 mg lycopene from tomato juice. *Represents significance p < 0.05.

Intervention with 20 mg of lycopene to the diet of subjects did not alter their total cholesterol, HDL-C or triglyceride  status. However, there were gender differences and the women had higher average levels of plasma triglycerides, total cholesterol and HDL-C than men (Figure 1). The higher cholesterol levels for women compared to men in this study were not unusual since women in this age range often have higher cholesterol levels than men, a phenomenon related to decreased estrogen production [24,25]. In this study, menopausal information was not recorded.

There was a significant positive correlation between each pair of total cholesterol and MDA and MDA and FRAP and between HDL-C and MDA and HDL-C and GPX. A significant negative correlation was found between triglycerides and GPX (Table 2).

**Table 2:** Spearman's Correlation Coefficients between antioxidant tests of malondialdehyde (MDA) ferric reducing ability of plasma (FRAP), and plasma glutathione peroxidase (GPX) and cholesterol measurements.

Variable	MDA	FRAP	GPX
Total cholesterol	0.547**	0.325**	0.003
HDL-C	0.563**	0.059	0.294**
Triglycerides	0.219	0.037	-0.229*
MDA	.	0.474**	0.180
FRAP	0.474**	.	0.077

Correlations significant at the 0.05* and 0.01** level.

Because of the correlation between cholesterol concentrations and antioxidant analysis, a preliminary analysis of data was conducted to determine if cholesterol levels impacted antioxidant results. Subjects were separated into two groups based upon baseline concentrations of plasma triglycerides, total cholesterol and LDL-C above or below 200, 180 and 160, respectively. Five subjects, two men and three women, fit the criteria of moderately hypercholesterolemic (Table 3).

**Table 3:** Separation of subjects by baseline cholesterol levels from a watermelon and tomato juice lycopene intervention study, n = 5 for each group.

Cholesterol Group	Total Cholesterol mg/dl	Triglycerides mg/dl	HDL-Cmg/dl
1	229.3 ± 4.9	190.9 ± 12.8	59.1 ± 2.9
2	176.6 ± 2.5	129.7 ± 5.2	46.9 ± 2.1

Data represents mean ± SE

Analyses showed an interaction of cholesterol level × treatment period × treatment factor for MDA and FRAP analysis. Higher MDA and FRAP levels were found in the group having higher cholesterol levels compared to the other group (Table 4). No trend with cholesterol level and glutathione peroxidase was found.

**Table 4:** Total, triglyceride, high density lipoprotein (HDL-C cholesterol and antioxidant analysis of malondialdehyde (MDA), glutathione peroxidase (GPX) and ferric reducing ability of of plasma (FRAP) after 4 week lycopene depletion and three weeks of intervention with watermelon and tomato juice (20 mg lycopene/day). Subjects were separated into 2 groups based upon cholesterol levels (see Table 3).

Cholesterol Group	Analysis	Depletion		SE	Control		SE	Watermelon		SE	Tomato		SE
	Total Cholesterol (mg/dl)	220.9	±	9.5	223.4	±	7.9	224.6	±	8.2	233.6	±	6.2
	Triglycerides (mg/dl)	185.9	±	16.9	181.7	±	16.9	198.9	±	18.3	174.7	±	15.6
1	HDL-C (mg/dl)	56.9	±	5.15	58.65	±	4.31	58.00	±	5.96	59.38	±	4.55
	MDA (umol/L)	1.21	±	0.11	1.12	±	0.11	1.15	±	0.12	1.37	±	0.11
	GPX (umol/L)	2728	±	219	2728	±	222	2263	±	169	2574	±	187
	FRAP (umol/L)	831.6	±	24.9	871.7	±	26.7	900.9	±	25.2	861.6	±	23.4
	Total Cholesterol (mg/dl)	182.6	±	4.2	173.3	±	3.3	186.1	±	6.4	173.1	±	2.8
	Triglycerides (mg/dl)	129.4	±	8.3	128.7	±	7.6	189.4	±	4.9	135.4	±	10.7
2	HDL-C (mg/dl)	42.5	±	3.3	43.6	±	2.8	44.7	±	4.0	38.3	±	2.8
	MDA (umol/L)	0.53	±	0.03	0.56	±	0.04	0.48	±	0.04	0.54	±	0.03
	GPX (umol/L)	2129	±	151	2111	±	154	2292	±	168	2229	±	160
	FRAP (umol/L)	743.9	±	33.6	762.9	±	30.7	780.9	±	32.4	756.7	±	36.0

## Discussion

We found no improvement in the antioxidant status of healthy middle-aged adults supplemented with two lycopene-containing foods. In previous antioxidant studies, reduced lipid peroxidation was reported in subjects supplemented from one to four weeks with 5 to 45 mg lycopene containing tomato products [15,16,23,26]. However, in each of these studies, the diet was not controlled. When healthy elderly subjects in a diet controlled study were supplemented with 13.3 mg of tomato lycopene (LycoRed) for 12 weeks, lycopene intervention did not significantly change LDL oxidation, as measured by the rate of conjugated diene production [27].

The reports from lycopene intervention studies that measured FRAP activity are not in agreement. One study reported improvement in FRAP levels of plasma in subjects supplemented with tomato juice and olive oil [28], while two other tomato juice intervention studies reported no improvement in plasma antioxidant levels after lycopene supplementation as measured by Trolox equivalent antioxidant capacity (TEAC), radical trapping antioxidant parameter assay (TRAP), and FRAP [3,23]. Researchers in one study found that the FRAP assay was more accurate when measuring the antioxidant activity of water-soluble antioxidants [23]. They thought full expression of the antioxidant activity was not identified from lycopene in this assay since it is a lipophyllic compound. Curiously, both watermelon and tomato contain other water-soluble compounds that are reported to have antioxidant activity that reacts *in vitro *in the FRAP assay [9,29]. In this study, contribution of these water-soluble compounds in changes in plasma FRAP activity with either food intervention compared to the control was not found.

Unlike a previous report by Fuhrman et al., neither lycopene intervention with watermelon nor tomato affected cholesterol levels [19]. Differences in results may have been due to lycopene dosage level. In that study [19] the subjects were supplemented with 60 mg/day for three months, however diet was not controlled.

Fruits and vegetables are excellent sources of antioxidant compounds and the average American consumes only 1.5 and 3.1 servings per day [45]. In many of the studies where antioxidant protection with lycopene containing foods was reported, subjects consumed their normal diet that may or may not have met the recommended servings of fruits and vegetables [13,23,26,31,32]. Increasing fruit and vegetable consumption to 12 servings per day compared to 5.8 servings, without the addition of other diet interventions, reduced a biomarker of DNA oxidative damage (8-hydroxydeoxyguanosine) by 32% [33]. In a controlled trial where subjects were supplemented with tomato juice but restricted in total fruit and vegetable consumption and exposed to low levels of ozone, researchers found reduced DNA strand breaks compared to placebo controls [34]. Because this study controlled for other phytochemical containing fruits and vegetables, the DNA protection was attributed to tomato juice phytochemicals [34].

The positive correlation between total cholesterol and MDA antioxidant analysis has been reported in studies with hypercholesterolemic subjects compared to normocholesterolemic subjects [35,36]. The MDA assay measures lipid peroxidation products, and a higher level of lipids available to react with peroxidizing agents results in higher MDA values [36,37].

The trend correlating higher FRAP with higher cholesterol levels has not been previously reported. The significance of this trend is speculative, since the FRAP assay measures the oxidation and reduction potential of compounds based on the reduction of the ferric to ferrous iron [38], lipid peroxidation products may have contributed to the oxidation/reduction potential of the reaction.

## Conclusions

Long-term supplementation studies where diet is controlled will probably be necessary to identify the benefits provided by lycopene. There may be real health benefits associated with lycopene especially since it is stored in various tissues and exhibits strong antioxidant activity *in vitro *[8,10,39,40]. Also the body of epidemiological evidence points to the protection provided against cardiovascular disease and some cancers with lycopene containing foods [11,12,41,42]. Recent cancer intervention studies have reported beneficial effects on prostate cancer from lycopene food supplementation [43,44]. The health benefits associated with diets providing lycopene are most likely long-term. Therefore, the findings of the present study should not be interpreted as a lack of health benefits from regular consumption of lycopene-rich foods.

The interaction between cholesterol levels and antioxidant values needs more research. Contradictory findings of this study with other *ex vivo *antioxidant studies may be due to the cholesterol levels of subjects thus warranting further research.

## Competing interests

None declared.

## Note

Mention of trade names or commercial products in this article is solely for the purpose of providing specific information and does not imply recommendation or endorsement by the U.S. Department of Agriculture. All programs and services of the U.S. Department of Agriculture are offered on a nondiscriminatory basis without regard to race, color, national origin, religion, sex, age, marital status, or handicap. The article cited was prepared by a USDA employee as part of his/her official duties. Copyright protection under U.S. copyright law is not available for such works. Accordingly, there is no copyright to transfer. The fact that the private publication in which the article appears is itself copyrighted does not affect the material of the U.S. Government, which can be freely reproduced by the public.

## Authors' contributions

JKC: conception and design of the study, drafted the manuscript, BHA: design of study, editing, PLC:design of the study, statistical analysis, PPV: conception of study, editing, RAB: editing, technical assistance, BAC:conception of study, editing. All authors read and approved the final manuscript.
